# Circulating hematopoietic stem cell count is a valuable predictor of prematurity complications in preterm newborns

**DOI:** 10.1186/1471-2431-12-148

**Published:** 2012-09-17

**Authors:** Maciej Kotowski, Krzysztof Safranow, Miłosz P Kawa, Joanna Lewandowska, Patrycja Kłos, Violetta Dziedziejko, Edyta Paczkowska, Ryszard Czajka, Zbigniew Celewicz, Jacek Rudnicki, Bogusław Machaliński

**Affiliations:** 1 Department of General Pathology, Pomeranian Medical University in Szczecin, Powstancow Wlkp. 72, Szczecin 70-111, Poland; 2 Department of Biochemistry and Medical Chemistry, Pomeranian Medical University in Szczecin, Powstancow Wlkp. 72, Szczecin 70-111, Poland; 3 Department of Obstetrics and Gynecology, Pomeranian Medical University in Szczecin, Powstancow 3Wlkp. 72, Szczecin 70-111, Poland; 4 Department of Obstetrics and Perinatology, Pomeranian Medical University in Szczecin, Powstancow Wlkp. 72, Szczecin 70-111, Poland; 5 Department of Neonatology, Pomeranian Medical University in Szczecin, Powstancow Wlkp. 72, Szczecin 70-111, Poland

**Keywords:** Hematopoietic stem cells, Very small embryonic-like stem cells, Peripheral blood circulating stem cells, Blood testing, Complications of prematurity, Premature infants

## Abstract

**Background:**

The frequency of preterm labour has risen over the last few years. Hence, there is growing interest in the identification of markers that may facilitate prediction and prevention of premature birth complications. Here, we studied the association of the number of circulating stem cell populations with the incidence of complications typical of prematurity.

**Methods:**

The study groups consisted of 90 preterm (23–36 weeks of gestational age) and 52 full-term (37–41 weeks) infants. Non-hematopoietic stem cells (non-HSCs; CD45^-^lin^-^CD184^+^), enriched in very small embryonic-like stem cells (VSELs), expressing pluripotent (Oct-4, Nanog), early neural (β-III-tubulin), and oligodendrocyte lineage (Olig-1) genes as well as hematopoietic stem cells (HSCs; CD45^+^lin^-^CD184^+^), and circulating stem/progenitor cells (CSPCs; CD133^+^CD34^+^; CD133^-^CD34^+^) in association with characteristics of prematurity and preterm morbidity were analyzed in cord blood (CB) and peripheral blood (PB) until the sixth week after delivery. Phenotype analysis was performed using flow cytometry methods. Clonogenic assays suitable for detection of human hematopoietic progenitor cells were also applied. The quantitative parameters were compared between groups by the Mann–Whitney test and between time points by the Friedman test. Fisher’s exact test was used for qualitative variables.

**Results:**

We found that the number of CB non-HSCs/VSELs is inversely associated with the birth weight of preterm infants. More notably, a high number of CB HSCs is strongly associated with a lower risk of prematurity complications including intraventricular hemorrhage, respiratory distress syndrome, infections, and anemia. The number of HSCs remains stable for the first six weeks of postnatal life. Besides, the number of CSPCs in CB is significantly higher in preterm infants than in full-term neonates (p < 0.0001) and extensively decreases in preterm babies during next six weeks after birth. Finally, the growth of burst-forming unit of erythrocytes (BFU-E) and colony-forming units of granulocyte-macrophage (CFU-GM) obtained from CB of premature neonates is higher than those obtained from CB of full-term infants and strongly correlates with the number of CB-derived CSPCs.

**Conclusion:**

We conclude that CB HSCs are markedly associated with the development of premature birth complications. Thus, HSCs ought to be considered as the potential target for further research as they may be relevant for predicting and controlling the morbidity of premature infants. Moreover, the observed levels of non-HSCs/VSELs circulating in CB are inversely associated with the birth weight of preterm infants, suggesting non-HSCs/VSELs might be involved in the maturation of fetal organism.

## Background

The premature birth of infants is an important issue facing perinatologists and pediatricians, mostly in developed countries, where the scale of preterm births is constantly increasing [1]. Independent of the cause of preterm labour, premature infants of low birth weight (BW) reveal a high risk of complications, including brain injury (e.g. intraventricular hemorrhage; IVH), retinopathy of prematurity (ROP), neonatal respiratory distress syndrome (RDS), bronchopulmonary dysplasia (BPD), necrotizing enterocolitis (NEC), and anemia, resulting in psychomotor disability. Development and progression of these serious complications may result from hypoxia, infection, metabolic disorders, and a general lack of adaptation to the extrauterine environment [2].

Survival of premature infants has significantly improved in recent years due to better care, nutrition, ventilation strategies, and behavioral adaptations [3]. However, mortality and morbidity rates are still relatively high in this population [3]. Infants of the same gestational age (GA) living in intrauterine conditions gradually mature to extrauterine life and usually do not suffer from organ damage or dysfunctions. Advances in research on stem cell biology have provided a chance for elucidation of the role of these cells in normal human development as well as in pathophysiological conditions. Evidence has accumulated indicating that a portion of stem/progenitor cells (SPCs) circulates in the peripheral blood to maintain a balance between SPCs populations in various anatomic areas of the living organism [4]. The number of these cells increases during organ and tissue injury, e.g. heart infarct, stroke, heavy burns, or liver or skeletal muscle damage [4-7]. Recent reports suggest that stem cells may play a role in the process of the endogenous regeneration of damaged organs [8].

In light of these issues, we investigated the potential association between the number of circulating stem and progenitor cell populations and levels of the chemokine regulating SPCs migration with the incidence of complications typical of prematurity in a prospective study. To date, no study has been performed to evaluate circulating SPCs numbers in premature infants within the first six weeks of extrauterine life. This study identified the kinetics of representative and highly selected SPCs populations circulating in the peripheral blood of premature and full-term babies at different time points in order to provide data showing a possible association with prematurity complications. Based on recent reports, the surface phenotypes for non-HSCs and HSCs were defined in our study as CD45^-^lin^-^CD184^+^ and CD45^+^lin^-^CD184^+^ cells, respectively [5,6]. The former cell population phenotypically corresponds to very small embryonic-like stem cells (VSELs), that express markers of pluripotent SCs (e.g., Oct4, Nanog, and SSEA-4) [9]. We also analyzed circulating stem/progenitor cells defined as CD133^+^CD34^+^ and CD133^-^CD34^+^ cells in order to investigate more differentiated blood cells. Finally, the clonogenic proliferative capacity of CB-derived hematopoietic progenitor cells from premature and full-term neonates was evaluated.

## Methods

### Characteristics of study groups

Subjects were recruited from patients of the Pomeranian Medical University in Szczecin, born between October 2007 and May 2010. We enrolled 90 preterm infants born at less than 37 weeks (wks) (23–36 wks) of gestational age and 52 healthy full-term infants (≥37 wks) as controls. The following exclusion criteria were applied: major congenital or chromosomal abnormalities, cyanotic heart defects, chronic intrauterine hypoxia (defined as growth retardation or pathologies of placental perfusion), transfusion in the operating theatre, exchange transfusion, or missing parental consent. Each child’s birth weight, sex, GA, Apgar scores, and neonatal clinical course were documented during the infants’ hospitalization and for the outpatient controls during six weeks of prospective observations after birth.

In each case, intraventricular hemorrhage (IVH) was diagnosed by ultrasonography (usg). Brain Doppler usg examination of the anterior cerebral artery and color cerebral function monitoring were performed for each patient with IVH.

Retinopathy of prematurity (ROP) was diagnosed and classified according to the International Classification of Retinopathy of Prematurity (International Committee) [10]. ROP was diagnosed in preterm infants based on retinal neovascularisation (stage 3 or more).

Respiratory distress syndrome (RDS) was diagnosed based on the physical examination, blood gas tests, and chest X-ray analysis using Bomsel’s classification [11] followed by echocardiography. Additionally, microbial tests were performed to rule out infection and sepsis.

Bronchopulmonary dysplasia (BPD) was diagnosed based on requirement of oxygen supplementation at 36 weeks postmenstrual age (PMA) and abnormal findings on chest radiography [12-14].

Necrotizing enterocolitis (NEC) was diagnosed in neonates who fulfilled at least stage II (definite NEC) of modified Bell’s criteria [15].

Anemia necessitating red blood cell transfusion was considered in spontaneously breathing infants (regardless of GA) if the hematocrit level fell to <40%, 35%, or 30% in the first, second, or third and fourth week of life, respectively. In infants older than 4 weeks, anemia necessitating transfusion was considered when the hematocrit level fell to <25%. In infants who were being ventilated or had an acute illness, the hematocrit threshold for transfusion was five percent higher than that mentioned above [16].

Neonatal infection was diagnosed based on a physician-defined examination that included serum C-reactive protein (CRP) (≥15 mg/L) and procalcitonin (PCT) (>0.5 ng/mL) analyses and blood cultures. Since positive blood cultures were not obtained in all cases of clinically diagnosed infections, they are treated as “suspected infections”. Within at least one week before blood collection, the enrolled patients were free of blood transfusions.

The control group consisted of full-term babies with no systemic, inherited or metabolic disorders. The study adhered to the tenets of the Declaration of Helsinki and approval was obtained from the Local Research Ethics Committee of the Pomeranian Medical University in Szczecin. The parents gave written informed consent for their child’s involvement.

### Sample collection

Venous blood samples (10 mL of CB and ~1.5 mL of PB) were collected in EDTA tubes at the moment of delivery as well as at the end of the second and sixth week after delivery. An aliquot of each sample was centrifuged (2000 rpm, 4°C, 10 min) and the plasma was stored at −80°C until assayed for SDF-1. The rest of the sample was used for flow cytometry, immunofluorescence and qRT-PCR. For the study of the kinetics of blood cell populations, we analysed data in subgroups of 13 preterm (GA: 33.2 ± 3.1 wks, BW: 2352 ± 778 g) and 18 full-term infants (GA: 38.9 ± 1.2 wks, BW: 3462 ± 338 g) for whom all three blood samples (CB, PB from the second and sixth week) were available.

### Flow cytometry

**Figure 1:**
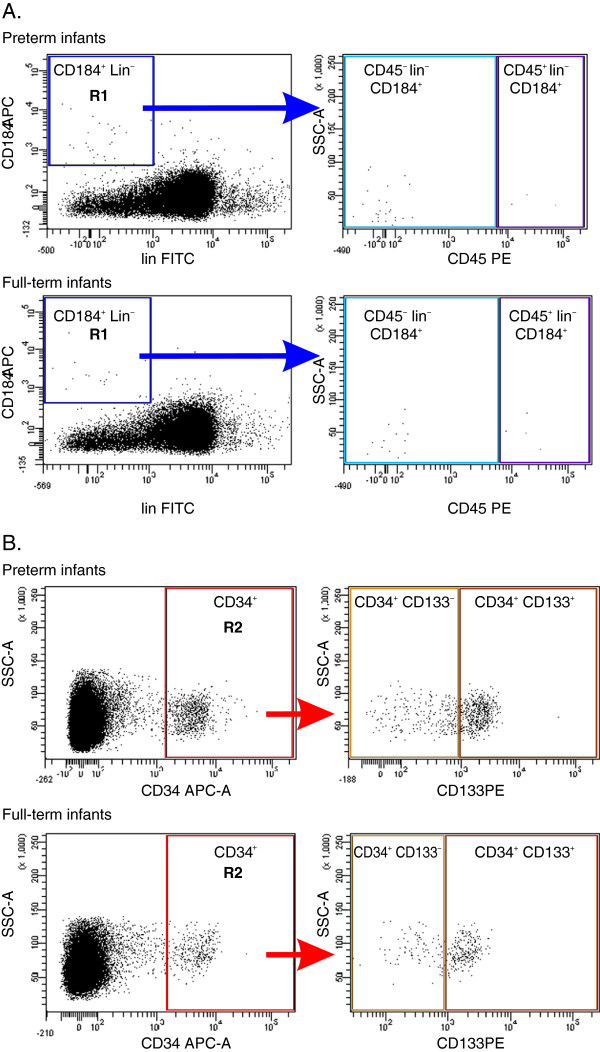
**A representative study of gating strategy for analyzing/sorting of non-HSCs/VSELs, HSCs, and CSPCs circulating in newborn PB by FACS.** Each colored gate illustrates a population of cells which expresses selected surface proteins. Panel **A**: Cytofluorimetric analysis of non-HSCs and HSC: CD184^+^lin^-^ cells were gated from a total fraction of immunofluorescence-stained nucleated cells from the PB of preterm and full term infants, then those cells from region R1 were further analyzed for CD45 expression; Panel **B**: Cytofluorimetric analysis of CSPCs (CD133^+^CD34^+^ and CD133^-^CD34^+^): CD34^+^ cells were gated from a total fraction of immunofluorescence-stained nucleated cells from the PB of preterm and full term infants, then those cells from region R2 were further analyzed for CD133 expression.

Whole CB or PB samples were lysed in BD PharmLyse Lysing Solution (BD Bioscences, San Jose, CA, USA) for 15 min at room temperature (RT) in the dark. The obtained suspension of CB or PB nucleated cells (NCs) was subjected to immunostaining procedures with murine anti-human monoclonal antibodies, as described previously [5,17]. For linage markers we used antibodies against: CD2 clone RPA-2.10, CD3 clone SK7, CD14 clone MϕP9, CD16 clone 3 G8, CD19 clone HIB19, CD24 clone ML5, CD56 clone NCAM16.2, CD66b clone G10F5, CD235a clone GA-R2 (all from BD Bioscences, San Jose, CA, USA). Flow cytometric analyses were performed using the LSRII instrument (BD, Biosciences, San Jose, CA, USA). Data were acquired with Cell Quest software (BD Biosciences, San Jose, CA, USA). Only freshly isolated cells were stained for flow cytometry and samples were run in the cytometer following the standard cleaning procedure. At least 10^5^ CB-derived and PB-derived cells with the appropriate ratio of forward scatter to side scatter were acquired, and all NCs were gated. In each case of CB, the collection of NCs was further extended to 2×10^6^ events per sample in order to increase enumeration rate of analyzed rare subpopulations of SCs, and thus augment the predictive value of the obtained results. The relative number of cells in each population was expressed as a percentage of the analyzed NCs after resting the number of total events found in the corresponding negative isotype control gate. The gating strategy for the analysis and sorting by FACS of different SC populations in this study is presented in Figure 1. 

### Isolation and immunofluorescence of cord/peripheral blood-derived SCs

The population of CD45^-^lin^-^CD184^+^ cells was sorted by multiparameter, live sterile cell sorting (BD FACSAriaIIu Cell-Sorting System, BD Biosciences, San Jose, CA, USA). Cell staining for all of the antigens was performed as described previously [17]. The sorted cells were permeabilized (0.1% Triton X-100, 10 min) and stained for β-III-tubulin, Nanog and Oct-4 antigens with anti-human monoclonal antibodies (Abcam, Cambridge, UK) for 1 h at RT, followed by incubation with a secondary antibody conjugated to Texas Red® (Vector Labs, Burlingame, CA, USA). The cells were subsequently fixed (3.7% paraformaldehyde) and their nuclei were stained with DAPI (Invitrogen, Paisley, UK). For fluorescence images, the BD Biosciences Pathway 855 bioimager (BD Biosciences, Rockville, MD, USA) was used.

### Real-time reverse transcriptase-polymerase chain reaction (qRT-PCR)

To analyze the mRNA levels for pluripotent (Oct-4, Nanog), early neural markers (β-III-tubulin), and oligodendrocyte lineage genes (Olig-1), total mRNA was isolated from CB cells using the RNeasy Mini Kit (Qiagen GmbH). Subsequently, the mRNA was reverse-transcribed using the First Strand cDNA Synthesis Kit (Fermentas International Inc., Burlington, ON, Canada). A quantitative assessment of mRNA levels was performed by using a Bio-Rad CFX96 Real-Time PCR Detection System (Bio-Rad Inc., Philadelphia, PA, USA). A 15 μL reaction mixture containing 7.5 μL iQ SYBR Green SuperMix (Bio-Rad Inc., Philadelphia, PA, USA) and 10 ng of complementary (c) DNA template, and 0.9 μM of forward and reverse primers for Oct-4, Nanog, β-III-tubulin, Olig-1 and β2-microglobulin (BMG) was used. The cDNA was amplified under the following conditions: 1 cycle at 50°C for 2 min and at 95°C for 10 min, followed by 40 cycles at 95°C for 15 s and at 60°C for 1 min. Relative quantification of mRNA expression was calculated by the comparative Ct method. The relative quantization value of the target, normalized to an endogenous control gene (BMG) and relative to a calibrator, was expressed as 2^-ΔΔCt^, where ΔCt = Ct of the target genes - Ct of the endogenous control gene, and ΔΔCt = ΔCt of the sample - ΔCt of the calibrator.

### Cell cultures

The CD34-positive cells (2 × 10^4^) were obtained by immunomagnetic beads from the CB of 44 preterm (GA: 33.3 ± 3.2 wks, BW: 2239 ± 640 g) and 24 full-term infants (GA: 39.2 ± 1.1 wks, BW: 3485 ± 404 g) using CD34 MicroBead Kit (Miltenyi Biotec Auburn, CA, USA) according to the manufacturer’s instructions. Next, the isolated CD34^+^ cells were resuspended in 0.4 mL of Iscove’s Modified Dulbecco’s Medium and mixed with 1.8 mL of the methylcellulose medium MethoCult HCC-4230 (StemCell Technologies Inc., Vancouver, BC, Canada) supplemented with L-glutamine and antibiotics. The appropriate recombinant human growth factors were added to the mixture as described previously [18]. The colonies were counted using an inverted microscope on day 11 for BFU-E and on day 14 for CFU-GM. Cultures developed from each blood sample were analyzed in quadruplicate.

### Plasma concentration of SDF-1

Levels of stromal-derived factor-1 (SDF-1) were measured using the commercially available high-sensitivity enzyme-linked immunosorbent assay (ELISA) Quantikine human immunoassay kits (R&D Systems, McKinley Place, MN, USA) according to the manufacturer’s protocol. The absorbance was read at 450 nm using an ELX 808 IU automated Microplate Reader (Bio-Tek Instruments Inc, Winooski, VT, USA). The results were analyzed using a quadratic loglog curve fit.

### Statistical methods

Differences in the values of the quantitative parameters were compared between groups by the Mann–Whitney test and between time points by the Friedman test. Fisher’s exact test was used for qualitative variables. Correlations between parameters were assessed using Spearman’s rank correlation coefficient (Rs). Univariate and multivariate logistic regression were performed to check the associations between selected parameters and prematurity complications. A p value of <0.05 was considered statistically significant.

## Results

### Characteristics of the clinical parameters

The characteristics of the clinical parameters, including maternal data and the incidence of complications in preterm and full-term infants are summarized in Table 1. The mean difference in gestational age at birth between the preterm and full-term groups of infants was 6.3 weeks. Consequently, preterm infants had a significantly lower mean birth weight (2145 g vs. 3530 g) and Apgar score at 1 min (7.8 vs. 9.3). Both groups were matched for gender. The most frequent complications (>15% incidence) diagnosed in preterm infants during the six week prospective observation period were infections, IVH, RDS and anemia necessitating red blood cell transfusion. Out of the preterm infants, 42% had at least one of the seven complications analysed (Table 1). A subgroup of 13 infants with extremely low gestational age at birth (GA: ≤30 wks, mean ± SD: 26.8 ± 2.2 wks, BW: 1141 ± 370 g) had particularly high incidence of the complications (IVH-77%, RDS-77%, BPD-54%, ROP-38%, NEC-15%, infection-85%, anemia-100%). We did not observed the infant mortality during the whole period of the study.

**Table 1:** Characteristics of clinical parameters, maternal data and complications in preterm and full- term infants

**Parameter**	**Preterm infants N = 90**	**Full-term infants N = 52**	**p **^**a**^
	**Mean ± SD (Median) or n (%)**	**Mean ± SD (Median) or n (%)**	
Gestational age at birth [wks]	33.0 ± 3.0 (34)	39.3 ± 1.2 (39)	<0.0001
Body weight at birth [g]	2145 ± 628 (2175)	3530 ± 387 (3595)	<0.0001
Male gender	56 (62%)	24 (46%)	0.079
Cesarean section	35 (39%)	7 (14%)	0.0012
Apgar score at 1 min	7.8 ± 2.1 (8)	9.3 ± 1.3 (10)	<0.0001
IVH	19 (21%)	1 (2%)	0.0009
RDS	18 (20%)	0 (0%)	0.0002
BPD	7 (8%)	0 (0%)	0.048
ROP (stage 3 or more)	6 (7%)	0 (0%)	0.086
NEC (stage II or more)	3 (3%)	0 (0%)	0.30
Suspected infection	30 (33%)	1 (2%)	<0.0001
Anemia	16 (18%)	0 (0%)	0.0006
Any complication	38 (42%)	2 (4%)	<0.0001
Antenatal steroids	16 (18%)	0 (0%)	0.0006
Pre-eclampsia	4 (4%)	0 (0%)	0.30
Diabetes mellitus	0 (0%)	1 (2%)	0.37
Chorioamnionitis	6 (7%)	0 (0%)	0.086
Premature rupture of membranes	26 (29%)	8 (15%)	0.10

^**a**^ Mann–Whitney test for quantitative variables and Fisher’s exact test for qualitative variables.

### The number of circulating non-HSCs is inversely associated with the birth weight of preterm infants

**Figure 2:**
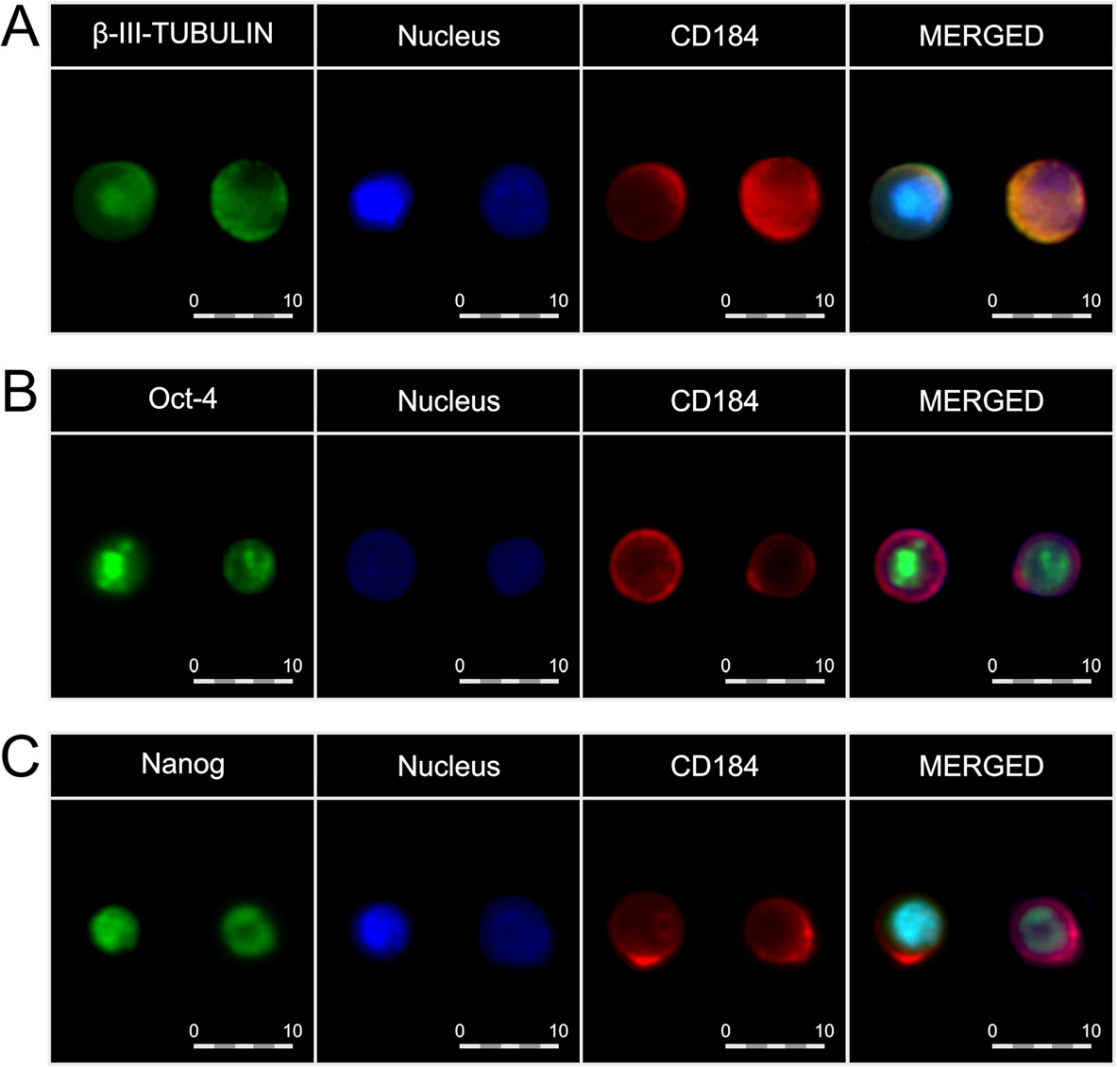
**Immunocytofluorescence of CB-derived non-HSCs. Individual images depict the expression of pluripotent/early neuronal markers, CD184, as well as nuclei in non-HSCs.** Merged images show coexpression of β-III-tubulin (panel **A**), Oct-4 (panel **B**), Nanog (panel **C**), and CD184, together with nuclei. Pseudocolors are assigned to each staining as follows: anti-β-III-tubulin, -Oct-4, and -Nanog – green, anti-CD184 – red, nuclei - blue. The cells were captured with × 40 objective magnification. The expression of each antigen was examined in cells in six independent experiments. Representative data are shown.

To analyze distinct stem cell types we selected CD45^-^lin^-^CD184^+^ and CD45^+^lin^-^CD184^+^ phenotypes that represent primitive, non-hematopoietic (non-HSC) and hematopoietic (HSC) stem cells, respectively [5,6,17]. Based on our previous reports, and in order to verify whether or not the population of non-HSCs comprise very small embryonic-like stem cells (VSELs), thus we evaluated in parallel the expression of markers common to pluripotent or undifferentiated cells by immunocytofluorescence and qRT-PCR in those cells. Hence, we sorted a population of non-HSCs from among the CB nucleated cells, and showed that these early cells expressed Oct-4, Nanog, and β-III-tubulin at the protein level, as revealed by immunofluorescence staining (Figure 2). Furthermore, in CB cells we noticed significant positive correlations between the number of non-HSCs and the expression of Oct-4 (Rs = +0.89, p = 0.019), Nanog (Rs = +0.89, p = 0.019), and β-III-tubulin (Rs = +0.90, p = 0.037) at the mRNA level, as revealed by qRT-PCR, whereas a correlation with Olig-1 expression did not reach statistical significance (Rs = +0.60, p = 0.21). No such positive correlations were found for HSC population. We therefore concluded that non-HSCs are the most primitive cells, enriched in VSEL SCs, from among the populations examined in our study, whereas HSCs could be defined as more differentiated cells. We used flow cytometry in order to estimate the number of examined SC populations in the CB of preterm and full-term infants. We found no significant differences in the number of CD45^–^lin^–^CD184^+^ or CD45^+^lin^–^CD184^+^ cells between the preterm and full-term infants (Table 2). There were no associations between the number of the above cells and the infants’ gender, type of delivery (physiological vs. cesarean section), age of gestation, or neonatal complications. However, in preterm infants we found a significant negative correlation between BW and the number of circulating non-HSCs/VSELs (Rs = −0.23, p = 0.033), although there was no correlation with the number of HSCs (Rs = +0.08, p = 0.44). These data revealed for the first time that the number of circulating non-HSCs/VSELs is inversely associated with the birth weight of preterm infants.

**Table 2:** Cord blood stem and progenitor cell populations and SDF-1 plasma concentrations in preterm and full-term infants

**Parameter**	**Preterm infants**		**Full-term infants**		**p **^**a**^
	**n**	**Mean ± SD (Median)**	**n**	**Mean ± SD (Median)**	
CD45^-^lin^-^CD184^+^ [%]	90	0.0294 ± 0.0319 (0.0180)	52	0.0209 ± 0.0201 (0.0135)	0.068
CD45^+^lin^-^CD184^+^ [%]	90	0.0016 ± 0.0032 (0)	52	0.0022 ± 0.0040 (0)	0.92
CD133^+^CD34^+^ [%]	90	0.349 ± 0.249 (0.313)	52	0.142 ± 0.088 (0.139)	<0.0001
CD133^-^CD34^+^ [%]	90	0.111 ± 0.092 (0.087)	52	0.037 ± 0.025 (0.0305)	<0.0001
SDF-1 [pg/mL]	90	1642 ± 488 (1562)	52	1727 ± 552 (1662)	0.35

^**a**^ Mann–Whitney test; n – number of measurements.

### The high number of circulating CB HSCs is strongly associated with a lower risk of IVH, RDS, infections, and anemia

To elucidate whether or not the concentration of SCs circulating in CB reveals relationship with the development of prematurity complications, we performed an extensive statistical analysis. As shown in Table 3, in newborns who developed IVH, RDS, infection, anemia, or any of the seven analyzed prematurity complications (in this subgroup, distinguished for statistical purposes, we grouped together preterm infants with at least one of the seven analyzed complications: IVH, RDS, BPD, ROP, NEC, infection, anemia) a significantly lower number of circulating CD45^+^lin^-^CD184^+^ cells was observed in comparison to infants without these complications. The incidence of any complication was significantly lower in the 42 preterm infants with the presence of at least 0.0010% HSCs in CB compared to 48 with <0.0010% HSCs (11/42 = 26% vs. 27/48 = 56%, p = 0.0054, OR = 0.28, 95% CI = 0.11-0.68). Of note, there was no association between the number of CD45^-^lin^-^CD184^+^ CB cells and any of the examined complications. Additionally, to check whether or not the association between the number of CD45^+^lin^-^CD184^+^ CB cells with the most frequent prematurity complications was independent from the other clinical factors, we performed logistic regression analysis (Table 4). Multivariate models with gestational age, type of delivery, Apgar score at 1^st^ minute, and the presence of at least 0.0010% HSCs in CB as independent variables showed number of CB HSCs ≥0.0010% were associated with a significantly lower risk of IVH, RDS, and infections, with borderline statistical significance (p = 0.092) for anemia. The association was also statistically significant for the development of any of the seven analyzed prematurity complications (OR = 0.28, 95% CI = 0.09-0.86, p = 0.024). Of note, in all multivariate models, the higher GA was associated with a lower risk of complications (OR from 0.25 to 0.68 per week). These results clearly indicate that the number of HSCs circulating in CB is an independent predictor inversely associated with the development of premature birth complications.

**Table 3:** Associations between prematurity complications and number of CB stem and progenitor cell populations in preterm infants

**Complication**	**Cord blood cell population**	**Complication absent [%]**	**Complication present [%]**	**p **^**a**^
		**Mean ± SD (Median)**	**Mean ± SD (Median)**	
IVH	CD45^–^lin^–^CD184^+^	0.0289 ± 0.0331 (0.0180)	0.0316 ± 0.0276 (0.0200)	0.41
	CD45^+^lin^–^CD184^+^	0.0020 ± 0.0035 (0.0010)	0.0004 ± 0.0008 (0)	0.014
	CD133^+^CD34^+^	0.342 ± 0.248 (0.306)	0.374 ± 0.258 (0.442)	0.52
	CD133^-^CD34^+^	0.100 ± 0.077 (0.087)	0.152 ± 0.126 (0.086)	0.26
RDS	CD45^–^lin^–^CD184^+^	0.0272 ± 0.0283 (0.0170)	0.0386 ± 0.0433 (0.0215)	0.13
	CD45^+^lin^–^CD184^+^	0.0020 ± 0.0034 (0.0010)	0.0003 ± 0.0006 (0)	0.0048
	CD133^+^CD34^+^	0.320 ± 0.238 (0.3053)	0.467 ± 0.265 (0.529)	0.027
	CD133^-^CD34^+^	0.095 ± 0.073 (0.083)	0.175 ± 0.128 (0.114)	0.019
BPD	CD45^–^lin^–^CD184^+^	0.0292 ± 0.0321 (0.0180)	0.0328 ± 0.0321 (0.0190)	0.76
	CD45^+^lin^–^CD184^+^	0.0017 ± 0.0033 (0)	0.0005 ± 0.0008 (0)	0.39
	CD133^+^CD34^+^	0.330 ± 0.244 (0.306)	0.570 ± 0.207 (0.544)	0.013
	CD133^-^CD34^+^	0.101 ± 0.080 (0.086)	0.238 ± 0.132 (0.248)	0.0074
ROP	CD45^–^lin^–^CD184^+^	0.0290 ± 0.0319 (0.0180)	0.0356 ± 0.0341 (0.0188)	0.68
	CD45^+^lin^–^CD184^+^	0.0017 ± 0.0033 (0)	0.0003 ± 0.0004 (0)	0.20
	CD133^+^CD34^+^	0.344 ± 0.251 (0.310)	0.424 ± 0.220 (0.510)	0.35
	CD133^-^CD34^+^	0.106 ± 0.085 (0.087)	0.186 ± 0.152 (0.162)	0.32
NEC	CD45^–^lin^–^CD184^+^	0.0298 ± 0.0324 (0.0180)	0.0180 ± 0.0046 (0.0190)	0.95
	CD45^+^lin^–^CD184^+^	0.0017 ± 0.0032 (0)	0 ± 0 (0)	0.12
	CD133^+^CD34^+^	0.338 ± 0.246 (0.310)	0.662 ± 0.014 (0.670)	0.015
	CD133^-^CD34^+^	0.108 ± 0.091 (0.086)	0.214 ± 0.032 (0.211)	0.025
Infection	CD45^–^lin^–^CD184^+^	0.0308 ± 0.0352 (0.0180)	0.0268 ± 0.0244 (0.0190)	0.96
	CD45^+^lin^–^CD184^+^	0.0020 ± 0.0036 (0.0010)	0.0010 ± 0.0021 (0)	0.034
	CD133^+^CD34^+^	0.345 ± 0.264 (0.307)	0.358 ± 0.220 (0.324)	0.60
	CD133^-^CD34^+^	0.105 ± 0.089 (0.085)	0.123 ± 0.098 (0.092)	0.43
Anemia	CD45^–^lin^–^CD184^+^	0.0298 ± 0.0333 (0.0180)	0.0276 ± 0.0252 (0.0195)	0.95
	CD45^+^lin^–^CD184^+^	0.0019 ± 0.0034 (0.0010)	0.0005 ± 0.0011 (0)	0.029
	CD133^+^CD34^+^	0.336 ± 0.247 (0.310)	0.409 ± 0.260 (0.464)	0.28
	CD133^-^CD34^+^	0.098 ± 0.077 (0.084)	0.175 ± 0.126 (0.152)	0.031
Any complication	CD45^–^lin^–^CD184^+^	0.0284 ± 0.0298 (0.0180)	0.0308 ± 0.0350 (0.0185)	0.86
	CD45^+^lin^–^CD184^+^	0.0021 ± 0.0038 (0.0010)	0.0010 ± 0.0019 (0)	0.014
	CD133^+^CD34^+^	0.341 ± 0.250 (0.307)	0.359 ± 0.251 (0.319)	0.71
	CD133^-^CD34^+^	0.099 ± 0.077 (0.085)	0.129 ± 0.107 (0.087)	0.30

^**a**^ Mann–Whitney test.

**Table 4:** Univariate and multivariate logistic regression models for the association of the presence of ≥0.0010% HSCs in CB with risk of the most frequent complications in preterm infants

**Logistic regression model**	**Complications**	**IVH**		**RDS**		**Infections**		**Anemia**		**Any complication**	
	**Independent variables**	**OR (95% CI)**	**p**	**OR (95% CI)**	**p**	**OR (95% CI)**	**p**	**OR (95% CI)**	**p**	**OR (95% CI)**	**p**
univariate	HSCs ≥ 0.0010%	0.23 (0.07-0.78)	0.017	0.17 (0.04-0.65)	0.0085	0.28 (0.11-0.73)	0.0088	0.21 (0.05-0.80)	0.021	0.28 (0.11-0.68)	0.0047
multivariate	HSCs ≥ 0.0010%	0.23 (0.05-0.997)	0.046	0.10 (0.01-0.86)	0.033	0.32 (0.10-1.01)	0.048	0.06 (0.002-1.67)	0.092	0.28 (0.09-0.86)	0.024
	Gestational age (wks)	0.68 (0.52-0.88)	0.0035	0.45 (0.30-0.68)	0.0002	0.65 (0.49-0.84)	0.0011	0.25 (0.10-0.61)	0.0021	0.54 (0.39-0.75)	0.0002
	Cesarean section (yes vs. no)	0.47 (0.12-1.94)	0.29	0.76 (0.14-4.13)	0.75	1.93 (0.63-5.93)	0.24	0.36 (0.03-4.59)	0.42	1.43 (0.46-4.40)	0.53
	Apgar score at 1 min (points)	0.80 (0.56-1.16)	0.23	0.99 (0.63-1.57)	0.96	0.96 (0.69-1.32)	0.79	0.93 (0.51-1.69)	0.81	1.03 (0.74-1.43)	0.87

### The population of circulating HSCs remains stable for the first six weeks of human life

**Figure 3:**
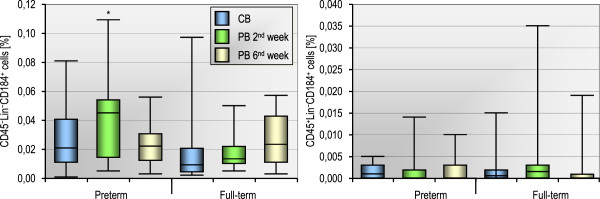
**The number of CD45**^**-**^**lin**^**-**^**CD184**^**+ **^**(non-HSCs) and CD45**^**+**^**lin**^**-**^**CD184**^**+**^** (HSCs) blood stem cell populations counted in CB at birth as well as in PB two and six weeks after birth in 13 preterm and 18 full-term infants.** *p < 0.05 for comparison between preterm and full-term infants (Mann–Whitney test). No statistically significant changes were observed within each group (p > 0.15, Friedman test).

In order to study the kinetics of the changes in the numbers of stem cells circulating in PB after birth, we prospectively analyzed the cell populations in the second and sixth week of extrauterine life, both in preterm and full-term infants. Figure 3 presents the numbers of two different blood SC populations (counted in CB at birth as well as in PB two and six weeks after birth) in preterm and full-term infants. We found no significant changes in the number of non-HSCs/VSELs and HSCs in CB compared to PB. However, two weeks after birth the number of PB non-HSCs/VSELs was significantly higher in preterm than in full-term infants (p = 0.018), although the difference was insignificant six weeks after birth (p = 0.68). Interestingly, we also observed a correlation between the number of HSCs in CB and in PB six weeks after birth, both in preterm (Rs = +0.89, p < 0.0001) and full-term infants (Rs = +0.66, p = 0.0028). This suggests that the HSC population is stable in the blood of preterm and full-term infants and that the number of these cells in CB at birth is a strong predictor of the cell number in PB six weeks later. This phenomenon may partly explain the protective effect of CD45^+^lin^-^CD184^+^ cells against prematurity complications (Table 4), because their low pool in CB at birth is associated with a long-term HSC deficit which lasts at least six weeks after delivery – the period when most complications develop.

Since stem cell migration is mediated by chemokines and other regulatory molecules, we studied changes in plasma concentrations of SDF-1, the most important chemotactic factor, critical for stem cell motility, homing, and mobilization [19]. We found no significant differences in SDF-1 concentrations in CB between preterm and full-term infants (Table 2). Moreover, SDF-1 levels in CB were not significantly associated with preterm infant gender, type of delivery, or any of the analyzed prematurity complications, and it did not correlate with the number of any CB cell population (data not shown). Nevertheless, in preterm infants, the plasma level of SDF-1 was significantly higher two weeks after delivery compared to cord blood (p = 0.011), whereas the difference was insignificant six weeks thereafter (p = 0.086). Furthermore, we noticed a strong correlation between CB SDF-1 levels and the number of non-HSCs/VSELs two weeks after delivery (Rs = +0.86, p = 0.014); however, this was statistically insignificant six weeks after delivery (Rs = +0.36, p = 0.43). Together these results suggest that SDF-1 may influence the circulating postnatal non-HSC/VSELs population in preterm infants.

### Premature infants contain a larger pool of progenitor cells circulating in their cord blood compared to full-term babies

To expand our study, we also measured the number of more restricted circulating stem/progenitor populations such as CD133^+^CD34^+^ and CD133^-^CD34^+^ cells that may contribute to tissue homeostasis by replenishing the lineage-specific hematopoietic cells. As shown in Table 2, preterm babies revealed in CB significantly higher numbers of CD133^+^CD34^+^ and CD133^-^CD34^+^ CSPCs than full-term infants (p < 0.0001). We also took into consideration the GA and evaluated whether or not this parameter could determine the number of CSPCs in CB. As revealed, a subgroup of 13 infants with extremely low GA at birth (≤30 weeks) had an even higher number of CD133^-^CD34^+^ cells than the remaining preterm infants (0.193 ± 0.131 vs. 0.098 ± 0.076%, p = 0.015). Nonetheless, no significant correlations between body weight at birth and CD133^+^CD34^+^ (Rs = −0.02, p = 0.85) or CD133^-^CD34^+^ cells (Rs = −0.09, p = 0.41) were found in preterm infants.

**Figure 4:**
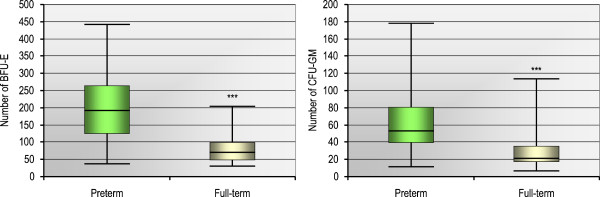
**Clonogenic growth efficiency of CD34-positive cells obtained from the CB of 44 preterm or 24 full-term infants.** Formation of burst-forming unit-erythroid (BFU-E) or colony-forming unit-granulocyte-monocyte (CFU-GM) was assessed in clonogenic in vitro assays. Colony counts are expressed in absolute values per 2 × 10^4^ plated cells and represent the median [min–max] from all the performed assays in each group. ***p < 0.001 for comparison between preterm and full-term infants (Mann–Whitney test).

Furthermore, we performed the clonogenic assays to verify whether or not the CD133^+^CD34^+^ and CD133^-^CD34^+^ CSPCs represent hematopoietic progenitors as similar phenotype might involve also endothelial progenitors to some extent [20]. Likewise, we found that the numbers of clonogenic BFU-E and CFU-GM were significantly higher in preterm infants compared to full-term infants (191 ± 91 vs. 78 ± 45, p < 0.0001 for BFU-E and 61 ± 34 vs. 33 ± 28, p < 0.0001 for CFU-GM, Figure 4). What is more, in preterm infants both BFU-E and CFU-GM colony numbers were strongly positively correlated with the number of CD133^+^CD34^+^ and CD133^-^CD34^+^ CSPCs, regardless of the difference between the two analyzed progenitor subpopulations. Of note, no correlation was observed between clonogenic growth and CD45^-^lin^-^CD184^+^ or CD45^+^lin^-^CD184^+^ CB cells, as well as the GA or SDF-1 plasma level in preterm infants (Table 5).

**Table 5:** Spearman rank correlation coefficients (Rs) for the association between cord blood-derived stem and progenitor cell populations and clonogenic potential in preterm infants (n = 44)

**Parameter**	**BFU-E**		**CFU-GM**	
	**Rs**	**p**	**Rs**	**p**
Gestational age	−0.16	0.29	−0.09	0.57
CD45^–^lin^–^CD184^+^	−0.08	0.62	−0.05	0.74
CD45^+^lin^–^CD184^+^	−0.04	0.79	+0.18	0.24
CD133^+^CD34^+^	+0.67	<0.0001	+0.51	0.0005
CD133^-^CD34^+^	+0.72	<0.0001	+0.52	0.0003
SDF-1	−0.26	0.092	0.00	0.98

BFU-E - number of erythrocyte burst-forming units.

CFU-GM - number of granulocyte–macrophage colony-forming units.

### The number of circulating progenitor cells firmly depends on gestational age of the infants

To elucidate whether or not the pool of CB-derived hematopoietic progenitors determines further development of premature complications in infants, we performed univariate and multivariate statistical analyses. The number of CD133^+^CD34^+^ and CD133^-^CD34^+^ cells circulating in CB was higher in preterm infants who developed RDS, BPD and NEC (Table 3), whereas anemia was associated only with a higher number of CD133^-^CD34^+^ CB cells. However, multivariate logistic analysis that was additionally adjusted for GA revealed that the higher number of CD133^+^CD34^+^ and CD133^-^CD34^+^ cells in CB is not an independent predictor of any of the prematurity complications (data not shown).

**Figure 5:**
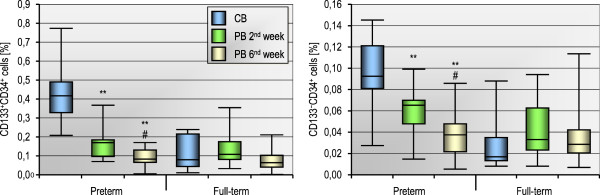
**The number of CD133**^**+**^**CD34**^**+ **^**and CD133**^**-**^**CD34**^**+**^** blood cells counted in CB at birth as well as in PB two and six weeks after delivery in 13 preterm and 18 full-term infants.** **p < 0.01 for comparison between PB in 2^nd^ and 6^th^ week vs CB. ^#^p < 0.05 for PB in 6^th^ week vs PB in 2^nd^ week (Mann–Whitney test).

Finally, to examine the changes in numbers of CSPCs in blood after birth, we counted the PB-derived progenitors in the second and sixth week after delivery in both groups of infants. As shown in Figure 5, the number of CD133^+^CD34^+^ and CD133^-^CD34^+^ cells significantly decreased (p < 0.01) in preterm infants in two and six weeks after delivery, reaching values similar to those observed in full-term infants, in whom the number of these cells was stable for the examined period of time. Since GA of full-term infants at birth was on average 6.3 weeks greater than that of preterm infants (Table 1), the PB taken from preterm babies in six weeks after delivery corresponds to the same GA as CB collected from full-term newborns. Thus, the kinetic profiles of CD133^+^CD34^+^ and CD133^-^CD34^+^ CSPCs suggest that their numbers in CB and PB physiologically decrease until about the 36^th^ week of GA, and are stable thereafter.

## Discussion

Preterm delivery is one of the most important factors of neonatal mortality and morbidity throughout the world. Recently, the incidence of perinatal death has considerably decreased as neonatological care has improved. However, managing morbidity after preterm labour is still a critical issue [21]. One of the current major issues in perinatal medicine is the search of valuable and early indicators of prematurity complications onset. Several recent retrospective studies were settled to find relationship between the blood characteristic markers or abnormalities and specific prematurity complication or an inflammatory process, however, not always giving the accurate results [22-25].

During prenatal development, various types of stem cells migrate, proliferate and differentiate to form tissues and organs. Tissue and peripheral blood SPCs pools are in dynamic equilibrium with each other, allowing stem cells to migrate from extravascular sites or marginal pools into the circulation and vice versa [26]. Whereas 37–42 weeks of gestation provide an optimal period of time for an infant’s maturation to extrauterine life, preterm birth deeply disturbs normal development. This prospective study was performed to elucidate the potential cause and effect relationship between the circulating SC populations and evolution of premature birth complications.

The absolute numbers of circulating non-HSCs/VSELs in PB are remarkably low (1–2 cells in 1 μL of blood under steady-state conditions). Similarly, circulating HSCs represent a very small fraction of PB cells, and thus special flow cytometry protocols have to be applied for identification of those highly restrictive SC populations [27]. We employed nuclear staining for detection of NCs present in blood samples and for exclusion of anucleated cellular debris from further analysis. Such a defined fraction of small NCs was further analyzed for CD45 and CD184 antigen expression, therefore two subpopulations, i.e. CD45^–^lin^–^CD184^+^ non-HSCs/VSELs and CD45^+^lin^–^CD184^+^ HSCs, could be distinguished [27]. Receptor CXCR4 (CD184) plays an important role in the mechanisms of HSCs migration and repopulation, in regard to the observation, that murine fetuses lacking this receptor (CXCR4-null model) have multiple defects that are lethal, including impaired BM hematopoiesis [28]. Recently, we were able, not only to confirm the presence of such highly restrictive SCs populations among blood leukocytes, but also to quantitatively determine the absolute numbers of these infrequent cells circulating in the blood of patients with various tissue/organ injuries and disorders [5,6,17]. Of note, the defined CD45^–^lin^–^CD184^+^ and CD45^+^lin^–^CD184^+^ SC populations have not been previously evaluated in relatively long-term prospective observation in PB of preterm infants.

This study demonstrated for the first time that the number of primitive non-HSCs circulating in CB is inversely associated with the birth weight of premature infants. Our observations imply that during the fetal stage of human life, primitive, undifferentiated stem cells circulate in the blood in a large number and contribute to organ/tissue formation. Gradually, the number of non-HSCs decreases and stabilizes along with the infant’s maturation. The characteristics of the actually analyzed non-HSCs correspond with very small embryonic-like SCs (VSELs) expressing pluripotent SC markers, described recently in CB by Kucia et al. [29], with a contribution by our research team. It has been demonstrated in mice that VSELs reveal pluripotency and are able to form all types of mature cells [30,31]. Furthermore, after the number of molecular analyses and *in vivo* experiments performed by Ratajczak et al., it has been recently proposed that human CB-derived VSELs correspond to the population of the most primitive HSCs circulating in the PB [32]. Therefore, if a small premature infant loses a substantial number of circulating non-HSCs, enriched in VSELs, together with secundines, this might well have negative clinical consequences for the infant’s development in the long-term. As we and others have recently reported, VSELs are mobilized in the PB of patients with ischemic stroke, myocardial infarction, or heavy burns, and this increase in the number of circulating cells is accompanied by elevated plasma levels of SDF-1 [5-7]. It has been hypothesized that these cells could attempt to regenerate the damaged tissue. Although we did not observe strict evidence of an analogous phenomenon in this study, however the number of non-HSCs/VSELs circulating in PB of premature infants was significantly greater than in full-term babies two weeks after delivery. At the same time point, SDF-1 plasma levels in preterm infants were considerably higher compared to the initial concentration of this chemokine at premature birth. Noteworthy, we noticed a strong correlation between CB SDF-1 levels and the number of non-HSCs/VSELs two weeks after delivery. Together, these observations may suggest that similar pathophysiological responses observed in stroke patients are noticed in small premature infants in an attempt to maintain PB-derived non-HSCs in relatively high concentrations. In order to clarify whether or not and how the undifferentiated non-HSCs/VSELs actively support regeneration in the first few weeks of human life after birth, or whether they remain largely in a dormant state, further studies are necessary. However, the differentiation of VSEL-SCs into human tissue-specific SCs (e.g. hematopoietic or neural) is not entirely elucidated and it is postulated that this process requires a relatively long period of time [32].

Clearly, the question arises as to whether or not a number of SCs circulating in CB is associated with the development of premature birth complications. As a first step towards addressing this issue, we sought to identify the highly purified populations of SCs with either durable or limited cause-and-effect relationships. Our results provide for the first time evidence that the number of HSCs circulating in CB is the independent predictor inversely associated with the development of premature birth complications. These include infections, anemia, IVH, and RDS, complications associated with blood and vascular systems origin, the development of which appears to be conditioned either directly (anemia, infections) or indirectly (other complications) by HSCs activity. Hematopoiesis is a complex, hierarchical process which involves the expansion and differentiation of a limited number of HSCs into multipotential and lineage-restricted progenitors, leading to the production of mature and functional blood cells [33]. HSCs, and their downstream precursors such as common lymphoid or myeloid progenitors that possess substantial clonogenic properties, are able to repopulate the whole medullary and extramedullary hematopoiesis in case of blood loss that, for example, may occur in case of IVH in preterm neonates [34]. Similarly, rapid rate of neonatal growth is one of the causes of anemia of prematurity that usually occurs during the first or second week of life in very small preemies. On the other side, iatrogenic blood loss secondary to sampling of blood for laboratory tests is nowadays the commonest cause of anemia of prematurity [35]. Relative but chronic deficiency of RBCs must be quickly compensated by increased proliferative activity of HSCs to reduce the potential biological effects of the RBCs scarcity in the body that might result in or might exaggerate the hypoxia-related complications in preterm newborns. Similarly, HSCs and their progenies are responsible for proper homeostasis maintenance of immune cells of myeloid and lymphoid lineage that protect the newborns against infections during the early stages of post-natal development. Thus, taken together, the number of active HSCs determines blood morphology and functions to effectively prevent both anemia and infections. However, it should be noted that association of HSCs with anemia lost significance in multivariate model and severe confounding by gestational age was observed. Nevertheless, there was no proof for other such confounding effects regarding associations of other complications with HSCs, since the associations retained statistical significance in multivariate analyses and respective OR values were similar in the uni- and multivariate logistic regression models.

What is more, HSCs also influence vascular homeostasis since the endothelial progenitor/precursor cell types are thought to be derived from the hematopoietic and vascular systems [36]. The concurrent emergence of hematopoietic and endothelial precursors in the embryonic yolk sac, as well as their overlapping patterns of gene expression, provides circumstantial evidence for the derivation of these cell types through a common progenitor, called hemangioblast, capable of giving rise to both endothelial and hematopoietic SCs at the earliest stages of hemato-endothelial differentiation [37]. The multifaceted modality of hemangioblast progenies, up to some extent, might be responsible for the fact, that circulating HSCs derived from bone marrow have been shown to participate in the normal and pathologic postnatal angiogenesis. Moreover, they were also able to induce neovascularization when transplanted into ischemic tissues [38]. Since, in such indirect way, HSCs could also correspond to the development of IVH or RDS in premature infants. As there is at least indirect association between vascular dysfunction and pathogenesis of RDS and IVH, these pathologies might be defined as ‘vascular-associated’ disorders [39,40].

Furthermore, we showed that the number of HSCs remains stable for six weeks after birth, the period when most of the abovementioned complications develop. This, in turn, at least partly explains the protective effect of HSCs against prematurity complications, since their low pool in CB at birth is associated with a long-term HSC deficit as evidenced by strong correlations between HSC percentages at birth and six weeks after birth. Of note, the number of circulating HSCs, unlike the non-HSCs/VSELs, did not increase in parallel with increased SDF-1 plasma level, detected two weeks after birth, in preterm infants. Our finding is similar to the observation described recently in patients with heavy burns [7]. In the light of recent reports, the involvement of other factors including small bioactive lipids that may direct mobilization and trafficking of SCs, should be also considered to determine precisely the conditions responsible for egress and trafficking of CXCR-4 positive HSCs [41]. Indeed, Bowie et al. [42] have recently reported that up to ~4 weeks post-partum BM-derived HSCs have higher cell-cycling rates than those from adult BM. They provide evidence that the chronologically younger HSCs produce considerable amounts of SDF-1, which stimulates these cells into cycle, and this may reflect the need for HSCs proliferation or self-renewal during periods of rapid body growth. Probably, the above observation made by Bowie et al. [42] would also explain the lack of direct association between circulating HSCs counts and post-natal SDF-1 levels in full-term and preterm infants found in our study. It is postulated that secretion of considerable amounts of SDF-1, in an autocrine manner by cycling post-natal HSCs, might interfere with HSC ability to respond to a chemotactic SDF-1 gradient, and so impede their trans-marrow trafficking, lodgment and retention in BM niches [42].

To the best of our knowledge, this is the first report clearly demonstrating that the human HSC population is associated with certain prematurity complications. Our results are in accordance with other clinical studies and suggest an important role of different circulating SC subpopulations in the development of preterm birth complications. Likewise, recent findings suggest a protective role of circulating progenitor cells in respiratory system disorder found in neonates born prematurely. The group of Qi et al. observed that RDS survivors had higher counts of CD34^+^ progenitor cells compared to non-survivors. Moreover, they observed that low concentrations of circulating CD34^+^ cells were correlated with a prolonged duration of mechanical ventilation of neonates with RDS, which can indicate the disease progression [43]. In a similar manner, it has recently been suggested that circulating hematopoietic cells may play an important vital role in repairing injured tissues and in disease progression, and thus differential hematopoietic cell populations may reveal a special paradigm as predictors for human morbidity. Great attention has recently been given to selected subsets of circulating CD34^+^ cells in patients with ischemic heart disease, as these cell populations might include those involved directly or indirectly in vascular repair. Likewise, it has been recently demonstrated that some cells, defined according to the International Society of Hematotherapy and Graft Engineering (ISHAGE) criteria as circulating hematopoietic progenitor cells (HPCs; CD34^+^CD45^dim^VEGFR2^-^ and CD34^+^CD45^dim^CD133^+^VEGFR2^-^), were reduced in patients presenting clinical signs of endothelial dysfunction, and thus it was postulated that the number of HPCs might be an independent predictor of morbidity from cardiovascular diseases and a marker of atherosclerotic disease progression [44]. Interestingly, these results are, to some extent, analogous to our data, in that the number of highly selected circulating HSCs is inversely correlated with the risk of the development of prematurity complications. Altogether, based on the above-mentioned data, we postulate that analysis of circulating HSC counts may be a valuable predictor for preterm birth-related morbidity.

A number of different circulating hematopoietic cell subsets described as CD34-positive cells were recently shown to be altered in neonates with prematurity complications, but without discrimination between more specific cell phenotype characteristics such as Lin^-^ or CD184^+^CD45^+^ and CD184^+^CD45^-^[43,45]. These observations were confirmed and extended in the present study. Especially, Lin^-^CD184^+^ cells were suggested to be more immature precursor cells of CD34-positive cells with a higher potency regarding peripheral tissue repair mechanisms [46,47]. Finally, in some instances, HSCs have been shown to contribute to the regeneration of chronically injured non-hematopoietic tissues. It is interesting to note that, according to recent critical reports, HSCs neither undergo transdifferentiation into cell types other than hematopoietic lineage cells nor structurally contribute to non-hematopoietic tissue regeneration on a significant scale [48,49]. However, after incorporation into injured tissue, HSCs might well produce and secrete humoral factors, creating a conducive microenvironment which promotes cell chemoattraction, survival and proliferation [50]. Likewise, circulating CB and early post-natal bone marrow HSCs have been previously reported not to home to the BM niches and, within the HSCs engrafting populations, ~95% were in the G0 phase (see [42] and references therein). These observations suggest that mobilized HSCs migrate from BM to the peripheral circulation where they indirectly contribute to organ regeneration and help to restore the integrity of extra-marrow tissues. It is worth noting that HSC transplantation has recently been used extensively for the treatment of numerous metabolic diseases in children [51]. Because of the variety of functions of HSCs in the human organism, their precise role in each disease condition may vary and should be carefully examined.

Previous studies have shown that hematopoietic progenitor cells express the CD34 surface antigen [52]. Likewise, CD34-positive cells represent a functionally primitive population of progenitor cells that seem to have a higher cloning efficiency and a very rapid proliferative response to cytokine stimulation [53]. Moreover, recent studies in bone marrow have also identified a CD133^+^ cell population, which is rare, undergoes self-renewal and differentiation and might represent stem/progenitor cell population [54]. Thus, we evaluated cell surface expression of CD34 and CD133 antigens, aiming to characterize the highly heterogenic circulating progenitor cell compartment in the blood of preterm infants and their relation with preterm morbidity. Our results showed that the percentage of CSPCs detectable in CB was significantly higher in preterm newborns compared with full-term fetuses. These results are in accordance with those of Haneline et al. [55] and Opie et al. [56], who reported significantly higher numbers of circulating progenitors in the CB of surviving preterm infants (23–32 wks of GA) and stillborn fetuses (< 24 wks) compared with the CB of mature newborns. In addition, we found that the number of CB-derived CD133^+^CD34^+^ and CD133^-^CD34^+^ cells was higher in preterm infants who developed prematurity complications such as RDS, BPD and NEC. Besides, anemia was associated only with a higher number of CD133^-^CD34^+^ CSPCs. However, a statistically significant correlation between the number of CSPCs and GA was also noticed and the associations of CSPCs with prematurity complications lost significance in multivariate analysis adjusted for gestational age.

In the present study, to identify hematopoiesis-related cells among SPCs circulating in cord blood, clonogenic assays were employed. Here, we found that the proliferative potential of BFU-E and CFU-GM was significantly higher in preterm infants than in full-term infants and colony numbers positively correlated with the number of CSPCs in CB. Our findings seem to reproduce the studies of Opie et al. [56], showing a decreased frequency of clonogenic precursors with advancing gestational age. This strongly supports the notion that detected CD133^+^CD34^+^ and CD133^-^CD34^+^ cells, circulating in CB, determines a strong clonogenic potential of hematopoietic origin, whereas other examined populations such as CD45^-^lin^-^CD184^+^ and CD45^+^lin^-^CD184^+^ cells are not directly associated with clonogenicity, what might indicate their stem derivation and more quiescent state. Finally, the differences in BFU-E and CFU-GM numbers between preterm and full-term infants were consistent with differences in the number of CD133^+^CD34^+^ and CD133^-^CD34^+^ CB cells between these groups (Table 2). The results obtained strongly indicate that analyzed subpopulations represent CB-derived hematopoietic progenitors.

Furthermore, our data seemed to show significant changes in the numbers of CSPCs in blood after birth. The number of CD133^+^CD34^+^ and CD133^-^CD34^+^ cells in PB gradually decreased during the first six weeks after birth until the quantities were similar to those detected in full-term infants. As the observed decrease in the number of CSPCs runs in parallel with advancing GA only in the premature infants group, this could indicate that the quantities of hematopoietic progenitors decrease physiologically until the 36^th^ week of GA, and are stable thereafter.

## Conclusion

We showed that HSCs circulating in CB, are markedly associated with the development of premature birth complications. The present findings of the interdependence between complications related to premature birth and the number of circulating SCs offer an exciting approach for further investigations into the pathophysiological processes underlying the development of the most common prematurity disorders. Thus, HSCs ought to be considered as an attractive and potential target for further research as they may be relevant for controlling the morbidity of premature infants. Efficient prevention and treatment of these conditions remains a priority in medicine. Regardless of further developments in therapeutic strategies based on improvement of the neonatal care system, the profound understanding of pathophysiological mechanisms responsible for the development of pathological changes in the course of prematurity complications is particularly important for their effective treatment. Only a full explanation of all aspects of this process can contribute to a real improvement in prevention and therapy of such disorders.

## Abbreviations

BMG: β2-microglobulin; BPD: Bronchopulmonary dysplasia; CB: Cord blood; CSPCs: Circulating stem/progenitor cells; HSCs: Hematopoietic stem cells; IVH: Intraventricular hemorrhage; NEC: Necrotizing enterocolitis; non-HSCs: Non-hematopoietic stem cells; PB: Peripheral blood; qRT-PCR: Quantitative reverse transcriptase polymerase chain reaction; RDS: Respiratory distress syndrome; ROP: Retinopathy of prematurity; Rs: Spearman’s rank correlation coefficient; SCs: Stem cells; SDF-1: Stromal-derived factor-1; SPCs: Stem/progenitor cells; VSEL SCs: Very small embryonic-like stem cells.

## Competing interests

The authors declare that they have no competing interests.

## Authors' contributions

Dr. MK blood sample collection and flow cytometry analysis. Dr. KS statistical analysis and writing of the manuscript. Dr. M P. K data analysis, figure preparation and manuscript revision. Dr. JL blood sample collection, analysis of clinical history of newborns. Dr. P K visualization of non-HSCs on Pathway Bioimager System. Dr. V D ELISA test analysis. Dr. E P flow cytometry and qRT-PCR analysis. Prof. R C supervision of the umbilical cord blood collection. Prof. Z C supervision of the umbilical cord blood collection. Prof. J R pediatric evaluation of the neonates. Prof. B M conception of the work, supervision and writing of the manuscript. All authors read and approved the final manuscript.

## Funding

This work was supported by European Union structural funds – Innovative Economy Operational Program POIG.01.01.02-00-109/09-00.

## Pre-publication history

The pre-publication history for this paper can be accessed here:

http://www.biomedcentral.com/1471-2431/12/148/prepub
